# Embolization of Middle Meningeal Artery in Patients with Chronic Subdural Hematoma: A Systematic Review and Meta-Analysis of Randomized-Controlled Clinical Trials

**DOI:** 10.3390/jcm14092862

**Published:** 2025-04-22

**Authors:** Nikolaos M. Papageorgiou, Lina Palaiodimou, Konstantinos Melanis, Aikaterini Theodorou, Maria-Ioanna Stefanou, Panagiota-Eleni Tsalouchidou, Pinelopi Vlotinou, Lampis C. Stavrinou, Efstathios Boviatsis, Georgios Magoufis, Marios Themistocleous, Amrou Sarraj, Vijay K. Sharma, Nitin Goyal, Georgios Tsivgoulis

**Affiliations:** 1 Second Department of Neurology, “Attikon” University Hospital, School of Medicine, National & Kapodistrian University of Athens, 12462 Athens, Greece; 2 Department of Neurology & Stroke, Eberhard-Karls University of Tübingen, 72074 Tübingen, Germany; 3 Epilepsy Center Hessen, Department of Neurology, Philipps University Marburg, 35037 Marburg, Germany; 4 Department of Occupational Therapy, School of Health and Welfare Sciences, University of West Attica, 12243 Athens, Greece; 5 Second Department of Neurosurgery, “Attikon” University Hospital, School of Medicine, National & Kapodistrian University of Athens, 12462 Athens, Greece; 6 Interventional Neuroradiology Unit, Metropolitan Hospital, 18547 Piraeus, Greece; 7 Interventional Radiology Unit, Second Department of Radiology, ‘Attikon’ University General Hospital, National & Kapodistrian University of Athens, 12462 Athens, Greece; 8 Neurosurgical Department, Pediatric Hospital of Athens ‘Agia Sophia’, 11527 Athens, Greece; 9 Department of Neurology, University Hospitals Cleveland Medical Center, Cleveland, OH 44106, USA; 10 Division of Neurology, National University Hospital, Yong Loo Lin School of Medicine, National University of Singapore, Singapore 119077, Singapore; 11 Department of Neurology, University of Tennessee Health Sciences Center, Memphis, TN 38163, USA; 12 Neurosurgery, Semmes Murphey Foundation, Memphis, TN 38120, USA

**Keywords:** middle meningeal artery, subdural hematoma, hematoma thickness, functional outcome, mortality, randomized-controlled clinical trial, systematic review, meta-analysis

## Abstract

**Background:** Chronic subdural hematoma (cSDH) is a common neurosurgical condition, particularly among elderly patients. Middle meningeal artery (MMA) embolization has emerged as a minimally invasive adjunctive treatment aimed at reducing recurrence. However, its comparative efficacy and safety remain under investigation. **Methods:** In this systematic review and meta-analysis, randomized-controlled clinical trial (RCT) data evaluating MMA embolization combined with best medical therapy (BMT) versus BMT alone in adult patients with symptomatic cSDH were pooled. The primary efficacy outcome was recurrence or progression of hematoma at follow-up. Secondary efficacy outcomes included good functional outcome [modified Rankin Scale (mRS) score ≤ 2], independent ambulation (mRS score ≤ 3), and hematoma thickness at follow-up. The primary safety outcome was all-cause mortality. Procedure-related complications were assessed as a secondary safety outcome. **Results:** Six RCTs were included, comprising 760 patients treated with MMA embolization and 788 patients treated with BMT alone. MMA embolization significantly reduced recurrence compared to BMT alone (RR: 0.50; 95% CI: 0.37–0.69; six studies; I^2^ = 0%; number-needed-to-treat = 13). No significant differences were observed in good functional outcome (RR: 1.01; 95% CI: 0.97–1.05; three studies; I^2^ = 0%), independent ambulation (RR: 1.01; 95% CI: 0.99–1.04; three studies; I^2^ = 0%), or hematoma thickness at follow-up (SMD: −0.1; 95% CI: −0.3 to 0; four studies; I^2^ = 42%). All-cause mortality was similar between the two groups (RR: 1.01; 95% CI: 0.42–2.40; five studies; I^2^ = 44%). The pooled rate of procedure-related adverse events in the MMA embolization-group was 1% (95% CI: 0–3%; two studies; I^2^ = 35%). **Conclusions:** MMA embolization significantly reduced cSDH recurrence when used as an adjunct to BMT. However, it did not demonstrate a significant impact on functional outcomes or mortality in this meta-analysis. Further research is needed to identify patient subgroups that benefit most from MMA embolization and to evaluate its impact on cognitive function and quality of life using longer follow-up periods.

## 1. Introduction

Chronic subdural hematoma (cSDH) is one of the most common neurosurgical conditions, particularly affecting the elderly [1]. With a rapidly aging global population and increasing use of antithrombotic and anticoagulant medications, the incidence of cSDH is projected to rise sharply, potentially becoming the most frequent cranial neurosurgical disease by 2030 [2]. Characterized by the slow accumulation of blood between the dural and arachnoid membranes, cSDH often arises from minor trauma and manifests through a spectrum of neurological symptoms (from motor deficits and cognitive decline to altered consciousness) resulting in substantial morbidity [3].

Surgical evacuation, typically via burr-hole with drainage insertion or craniotomy, remains the mainstay for symptomatic cases. However, recurrence rates after surgery range from 8% to 30%, and reoperation is often required in up to 15% of patients [4,5]. Additionally, complications associated with surgical intervention, including infections, seizures, and functional deterioration, are particularly concerning in frail elderly patients with multiple comorbidities [6]. Middle meningeal artery (MMA) embolization, either as a primary treatment or as an adjunct to surgery, has emerged as a novel, minimally invasive therapeutic approach targeting the vascular supply of the outer membrane of the hematoma [7]. The rationale stems from growing evidence that cSDH is not simply a passive accumulation of blood but a dynamic inflammatory process driven by neovascularization and recurrent microhemorrhages [8]. By occluding the MMA, embolization aims to disrupt this pathological cycle, thereby reducing recurrence and promoting hematoma resolution, potentially improving functional outcomes and reducing the need for revision surgery [8].

Preliminary evidence from several observational studies has suggested that MMA embolization may reduce the risk of recurrence and improve outcomes in patients with cSDH [9,10,11,12]. These studies have reported promising results in both surgical and nonsurgical candidates, including elderly and coagulopathic patients. However, observational designs are inherently limited by issues such as confounding by indication, selection bias, and variability in treatment protocols and patient populations. As such, definitive conclusions regarding the efficacy and safety of MMA embolization have remained elusive. Recently, randomized-controlled clinical trials (RCTs) have attempted to address these limitations, providing the first rigorous evidence on the role of MMA embolization as an adjunct to standard therapy in cSDH. While the trials collectively indicate benefits in reducing recurrence and improving clinical outcomes, variations in trial design, timing of intervention, surgical approaches, and patient selection highlight the need for a comprehensive synthesis of the available evidence.

The objective of this systematic review and pairwise meta-analysis is to critically evaluate and quantify the efficacy and safety of MMA embolization combined with best medical therapy (BMT) compared to BMT alone in adult patients with symptomatic cSDH. By integrating data across different RCTs, we sought to clarify the role of MMA embolization in modern cSDH management and inform future clinical decision-making.

## 2. Methods 

### 2.1. Standard Protocol Approvals, Registrations, and Patient Consents 

The pre-specified protocol of the present systematic review and meta-analysis has been registered in the International Prospective Register of Ongoing Systematic Reviews PROSPERO (registration ID: CRD420251018825). The meta-analysis is reported according to the updated Preferred Reporting Items for Systematic Reviews and Meta-Analyses (PRISMA) guidelines [13]. This study did not require an ethical board approval or written informed consent by the patients according to the study design (systematic review and meta-analysis).

### 2.2. Data Sources, Searches, and Study Selection 

A systematic literature search was conducted according to the patient, intervention, comparison, and outcome (PICO) model [14] to identify available RCTs including adult patients with symptomatic cSDH (P: population) receiving MMA embolization together with BMT (I: intervention) versus BMT alone (C: control) and investigating the outcomes of interest as outlined below (O: outcome). cSDH was defined as a subdural collection of blood in the brain CT where 50% or more of the volume was either isodense or hypodense as compared to cortical gray matter. MMA embolization was characterized as the intra-arterial administration of an embolic agent, guided by digital subtraction angiography through the external carotid artery. BMT was defined as standard care, which could include surgical evacuation via a single or double burr-hole or craniotomy.

Observational cohort studies, non-controlled studies, case series, and case reports were excluded. Studies evaluating patients with non-symptomatic or acute SDH were not considered. Commentaries, editorials, and narrative reviews were also excluded. 

The literature search was performed independently by 4 reviewers (NMP, LP, KM, AT). The electronic databases MEDLINE and Scopus were searched, using search strings that included the terms “chronic subdural hematoma”, “middle meningeal artery”, and “embolization”. The complete search algorithm is provided in the Supplement. No language or other restrictions were applied. Our search spanned from the inception of each database to 25 March 2025. Furthermore, the reference lists of published articles and international conference abstracts were manually scrutinized to ensure the completeness of the bibliography. All retrieved studies were independently assessed by 4 reviewers (NMP, LP, KM, AT) and any disagreements were resolved after discussion with a fifth tie-breaking evaluator (GTs).

### 2.3. Quality Control, Bias Assessment, and Data Extraction 

Four reviewers (NMP, LP, MIS, PET) independently assessed quality control and bias assessment among eligible studies, employing the Cochrane Collaboration Risk-Of-Bias 2 tool (RoB 2) for RCTs [15]. Any disagreements were settled by consensus after discussion with the corresponding author (GTs). 

Data extraction was performed in structured reports, including study name, country, recruitment period, intervention and comparison characteristics, included patients, their baseline characteristics, and the outcomes of interest.

### 2.4. Outcomes

The primary efficacy outcome of interest was the recurrence or progression of the SDH on the target side at follow-up, as defined by each study. Good functional outcome [defined as modified Rankin Scale (mRS) score of 2 or less] [16], independent ambulation (defined as mRS-score ≤ 3) [16], and thickness of hematoma (in mm) at follow-up were assessed as secondary efficacy endpoints.

The primary safety outcome was all-cause mortality at follow-up. The procedure-related complications rate for MMA embolized patients was also assessed as a secondary safety outcome.

### 2.5. Statistical Analysis

For the pairwise meta-analysis, we calculated risk ratios (RR) with 95% confidence intervals (CIs) for dichotomous outcomes, i.e., the comparison of outcome events among patients receiving MMA embolization versus BMT. Standardized mean differences (SMD) with 95% CIs were calculated for continuous outcomes of interest (i.e., hematoma thickness). For every outcome of interest, the corresponding pooled proportion with 95% CI was calculated for each arm, after the implementation of the variance-stabilizing double arcsine transformation. All outcomes were assessed based on intention-to-treat analysis. For the primary efficacy outcome, the number needed to treat (NNT) was calculated using the formula: NNT = $\frac{1}{\left(1-RR\right)\times rate\;in\;BMT\;group}$ as previously described [17].

Comparison of the baseline characteristics to assess the balance between the two arms was performed using odds ratios for dichotomous variables and standardized mean differences for continuous variables. For studies reporting continuous outcomes in median values and corresponding interquartile ranges, we estimated the sample mean and standard deviation using the quantile estimation method [18]. The random-effects model (DerSimonian and Laird 1986) was used to calculate the pooled estimates [19].

The threshold for statistical significance for the above analyses was set at two-sided *p*-value of <0.05. Heterogeneity was assessed with the I^2^ and Cochran Q test. For the qualitative interpretation of heterogeneity, I^2^ values < 25%, between 25 and 50%, and >50% were considered to represent low, moderate, and significant heterogeneity, respectively. The significance level for the Q statistic was set at <0.1. Small-study effects, as a proxy for publication bias, were assessed when at least four studies were included in the analysis of the outcomes of interest, using both funnel plot inspection and the Egger’s linear regression test, with a two-sided *p*-value < 0.1 being considered statistically significant [20]. Prediction intervals (PI) were also calculated for all outcomes of interest to estimate the range of effects in future studies, as previously performed by our group [21]. The above statistical analyses were performed using the R software version 3.5.0 (package: meta) [22].

### 2.6. Data Availability Statement

All data generated or analyzed during this study are included in this article and its supplementary information files. 

## 3. Results

### 3.1. Literature Search and Included Studies

**Figure 1:**
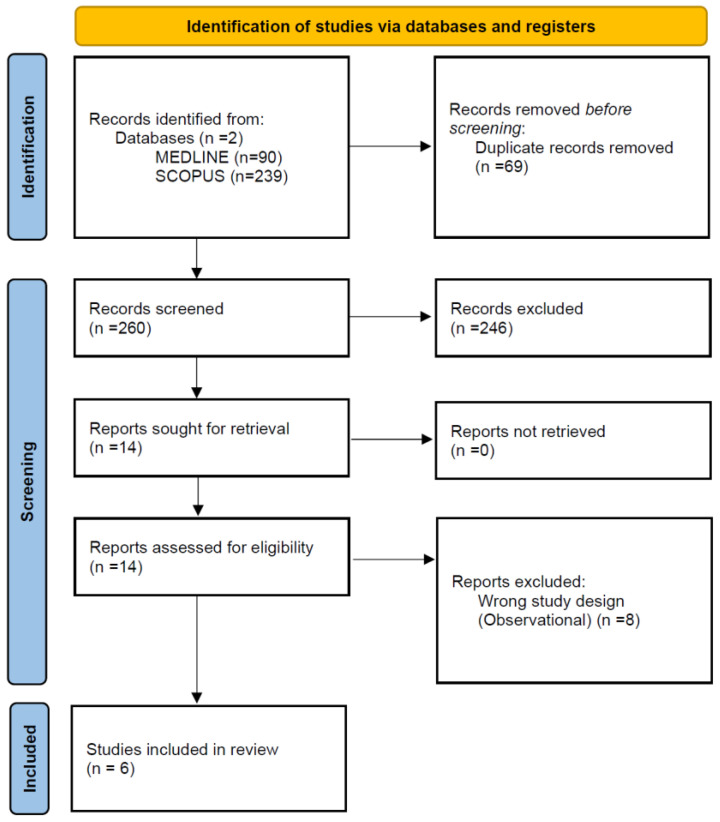
Flow chart of the systematic review.

The systematic database search yielded a total of 90 and 239 records from the MEDLINE and SCOPUS databases, respectively (Figure 1). After excluding duplicates and initial screening, we retrieved the full text of 14 records that were considered potentially eligible for inclusion. After reading the full-text articles, a further eight were excluded (Supplementary Table S1) [9,10,11,12,23,24,25,26]. Finally, we identified six eligible RCTs for inclusion in the systematic review and meta-analysis (Table 1) [27,28,29,30,31,32], comprising a total of 1548 patients with cSDH, receiving either MMA embolization together with BMT (*n* = 760; mean age 71.3 years; 28% female, 96% undergoing surgery) versus BMT alone (*n* = 788; mean age 71.2 years; 27% female, 95% undergoing surgery; Supplementary Figures S1–S3). In the intervention group, 27% of the patients were under antithrombotic medication (including anticoagulants or antiplatelets) at the time of index SDH and the mean baseline SDH thickness in brain CT was 19.7 mm. In the control group receiving BMT, 29% of the patients were under antithrombotic medication and the mean baseline SDH thickness was 19.7 mm (Supplementary Figures S4 and S5).

The included studies enrolled adult patients with confirmed chronic subdural hematoma, most commonly presenting with symptoms such as headache, gait instability, cognitive impairment, and focal neurological deficits attributable to the hematoma. Management strategies varied across studies. Different embolic materials were used in each study (Supplementary Table S2). In four RCTs, all participants underwent surgical evacuation (primarily through burr-hole drainage) as part of the standard of care [27,28,29,30]. In contrast, two trials allowed for individualized treatment decisions regarding surgical intervention, based on clinical and imaging criteria at the discretion of the treating physician [31,32]. Several trials applied specific procedural exclusions: for instance, wide craniotomy was explicitly excluded in two trials that focused solely on patients undergoing burr-hole drainage [31,32]. Additionally, the MAGIC-MT trial excluded patients with bilateral hematomas, thereby restricting its cohort to individuals with unilateral lesions [31]. Follow-up durations also differed among the studies, with two trials reporting outcomes at 180 and 125 days, respectively [29,32], whereas the remaining trials employed a 90-day follow-up period [27,28,30,31].

### 3.2. Quality Control of Included Studies

The assessment for the risk of bias in the included RCTs is presented in Supplementary Figure S6. More specifically, Debs et al. utilized a 1:1 consecutive randomization process resulting in high risk of bias in the randomization domain [29]. Furthermore, the EMBOLISE and the MAGIC-MT studies had some concerns regarding deviations from the intended intervention [30,31]. Three RCTs presented a high risk of bias, due to a rate of missing outcome data that exceeded 10% of the enrolled population [29,30,32]. All studies had some concerns in the measurements of outcomes mainly due to the unblinded assessment of the main outcome, as the embolization liquid is radio opaque and therefore it is visible on the CT scan. The risk of bias due to reporting of outcomes was low in all included studies. 

### 3.3. Quantitative Analyses

An overview of analyses for primary and secondary outcomes is summarized in Table 2.

**Figure 2:**
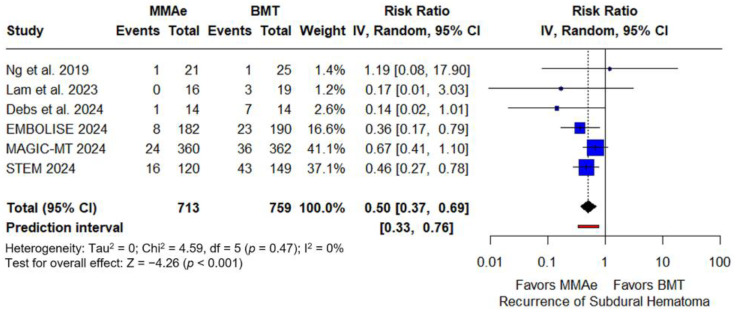
Forest plot presenting the risk ratio of recurrence of subdural hematoma (SDH) at follow-up among patients receiving middle meningeal artery embolization (MMAe) versus best medical treatment alone (BMT) [27,28,29,30,31,32]. Blue squares indicate study-specific estimates (size reflects study weight), with horizontal black lines showing 95% confidence intervals. The black diamond represents the pooled estimate, and the black dashed line marks the summary risk ratio. The black vertical line at 1.0 indicates no effect. The red horizontal line represents the prediction interval, reflecting the expected range of effect in future studies.

Regarding the primary efficacy outcome, patients receiving MMA embolization had significantly lower rates of SDH recurrence compared to those receiving BMT alone (RR: 0.50; 95% CI: 0.37–0.69; *p* < 0.001; PI: 0.33–0.76; 6 studies; I^2^ = 0%; p for Cochran Q = 0.47; Figure 2; Supplementary Table S3). The NNT of MMA embolization vs. BMT for cSDH was 13 (95% CI: 11–22).

**Figure 3:**
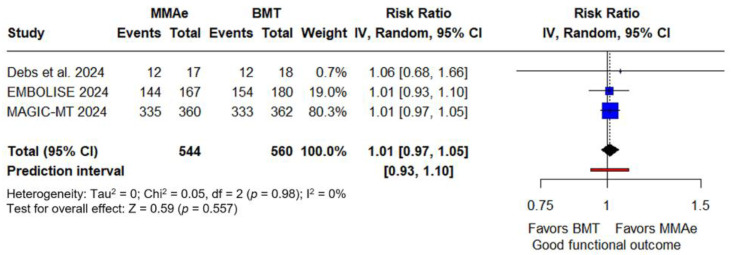
Forest plot presenting the risk ratio of good functional outcome at three months among patients receiving middle meningeal artery embolization (MMAe) versus best medical treatment alone (BMT) [29,30,31]. Blue squares indicate study-specific estimates (size reflects study weight), with horizontal black lines showing 95% confidence intervals. The black diamond represents the pooled estimate, and the black dashed line marks the summary risk ratio. The black vertical line at 1.0 indicates no effect. The red horizontal line represents the prediction interval, reflecting the expected range of effect in future studies.

**Figure 4:**
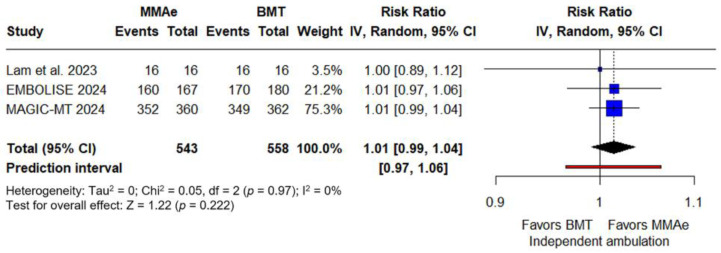
Forest plot presenting the risk ratio of independent ambulation at three months among patients receiving middle meningeal artery embolization (MMAe) versus best medical treatment alone (BMT) [28,30,31]. Blue squares indicate study-specific estimates (size reflects study weight), with horizontal black lines showing 95% confidence intervals. The black diamond represents the pooled estimate, and the black dashed line marks the summary risk ratio. The black vertical line at 1.0 indicates no effect. The red horizontal line represents the prediction interval, reflecting the expected range of effect in future studies.

**Figure 5:**
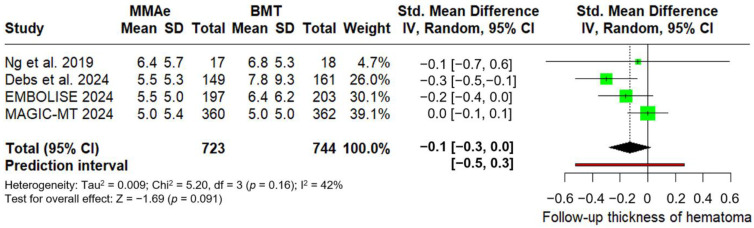
Forest plot presenting the standardized mean difference of subdural hematoma thickness (in mm) at follow-up among patients receiving middle meningeal artery embolization (MMAe) versus best medical treatment alone (BMT) [27,29,30,31]. Green squares indicate study-specific estimates (size reflects study weight), with horizontal black lines showing 95% confidence intervals. The black diamond represents the pooled estimate, and the black dashed line marks the summary risk ratio. The black vertical line at 0 indicates no effect. The red horizontal line represents the prediction interval, reflecting the expected range of effect in future studies.

Concerning secondary outcomes, similar rates of good functional outcome were noted for those receiving MMA embolization with BMT compared to those receiving BMT alone (RR: 1.01; 95% CI: 0.97–1.05; *p* = 0.557; PI: 0.93–1.10; three studies; I^2^ = 0%; p for Cochran Q = 0.98; Figure 3). Similarly, rates of independent ambulation did not differ between intervention and control group (RR: 1.01; 95% CI: 0.99–1.04; *p* = 0.222; PI: 0.97–1.06; three studies; I^2^ = 0%; p for Cochran Q = 0.97; Figure 4). In the follow-up brain CT, thickness of hematoma was similar between the two groups (SMD: −0.1; 95% CI: −0.3 to 0; *p* = 0.091; PI: −0.5 to 0.3; four studies; I^2^ = 42%; p for Cochran Q = 0.16; Figure 5).

**Figure 6:**
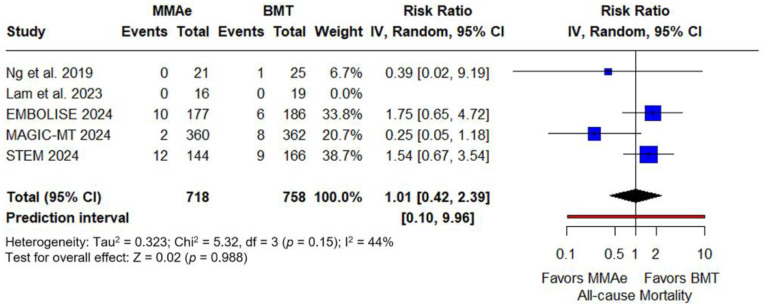
Forest plot presenting the risk ratio and prediction interval of all-cause mortality at follow-up among patients receiving middle meningeal artery embolization (MMAe) versus best medical treatment alone (BMT) [27,28,30,31,32]. Blue squares indicate study-specific estimates (size reflects study weight), with horizontal black lines showing 95% confidence intervals. The black diamond represents the pooled estimate, and the black dashed line marks the summary risk ratio. The black vertical line at 1.0 indicates no effect. The red horizontal line represents the prediction interval, reflecting the expected range of effect in future studies.

Regarding safety outcomes, all-cause mortality was similar between the MMA embolization and BMT groups (RR: 1.01; 95% CI: 0.42–2.39; *p* = 0.988; PI: 0.10–9.96; five studies; I^2^ = 44%; p for Cochran Q = 0.15; Figure 6). Procedure-related complications following MMA embolization were infrequent. Among the two trials reporting such events [31,32], adverse outcomes included facial nerve palsy, contrast-agent allergy, and ischemic stroke. No cases of blindness were reported. The pooled complication rate was 1% (95% CI: 0–3%; two studies; PI: 0–29%; I^2^ = 35%; p for Cochran Q = 0.21; Supplementary Figure S7).

The pooled proportions per arm for each outcome of interest are presented in Supplementary Table S4.

In the assessment of publication bias, no asymmetry was noted for any of the outcomes during inspection of the funnel plots (Supplementary Figures S8–S10).

## 4. Discussion

In this systematic review and meta-analysis, we included six RCTs comprising 1548 patients with cSDH and concluded that MMA embolization significantly reduces hematoma recurrence compared to BMT alone. Specifically, embolization was associated with a 50% reduction in recurrence risk, a finding that remained robust across studies with minimal statistical heterogeneity. However, no significant differences were observed in secondary outcomes, including good functional outcome, independent ambulation, residual hematoma thickness, or all-cause mortality.

The discrepancy between the reduced recurrence rate and the lack of difference in functional outcomes warrants further exploration. One possible explanation lies in the limitations of the mRS, a commonly used functional outcome measure [33] that may not fully capture the subtleties of cognitive and neurobehavioral impairment frequently observed in patients with cSDH. Given that cognitive deficits often predominate in this population [34], reliance on mRS, which primarily assesses mobility and independence, may underestimate the true benefit of embolization [35,36]. Moreover, motor deficits in cSDH may be subtle [37], and thus can be under-represented in mRS scoring. Notably, the MAGIC-MT trial employed a broader health-related quality of life assessment (EQ-5D-5L) [31], but similarly found no significant difference between groups, reinforcing the need for more sensitive and specific outcome measures in future trials. Another explanation for this discrepancy may be the timing of outcome assessment; functional recovery after SDH can be a slow process, especially considering the elderly population it usually pertains to. The three-month follow-up, common in most studies, may not be long enough to capture the full extent of functional improvement, even if recurrence is reduced. Moreover, timing relative to surgery may play a role as well; if embolization is performed after surgical evacuation, the initial surgery might have already set the trajectory for functional recovery. Embolization might prevent recurrence but not necessarily reverse pre-existing functional deficits. Notably, only three of the included RCTs reported functional outcomes using the mRS, limiting the strength of conclusions on functional recovery [28,30,31]. This further highlights the need for future trials to include comprehensive and sensitive measures of post-treatment neurological and cognitive function.

Regarding radiographic outcomes, follow-up hematoma thickness appeared marginally lower in the embolization group; however, this difference did not reach statistical significance. It is worth noting that the STEM trial [32], one of the largest of the included studies, did not report on hematoma thickness at follow-up, potentially limiting power in the analysis of this secondary outcome. Additional data from future RCTs are needed to clarify whether embolization meaningfully influences hematoma resolution on imaging.

Safety outcomes were also reassuring. Mortality rates were low and comparable between treatment arms, and adverse events directly attributable to the embolization procedure were rare. Given the lack of significant difference in all-cause mortality, NNT was not calculated for safety outcomes to avoid overinterpretation of non-significant findings. The favorable safety profile may be partly explained by the anatomical characteristics of the MMA, which plays a limited role in cerebral perfusion, and the use of the external carotid approach, minimizing the risk of intracranial ischemia. As embolization techniques continue to evolve and operator experience in endovascular techniques increases [38,39], complication rates may further decline, enhancing the safety and feasibility of this intervention in routine clinical practice.

An area of growing clinical interest is the potential role of MMA embolization as a primary standalone therapy for patients with cSDH, particularly those in whom surgical evacuation may be unnecessary or high-risk. It is important to note that all RCTs included in this meta-analysis investigated MMA embolization as an adjunct to BMT, which, in more than 95% of cases, included surgical evacuation. As such, the current analysis does not evaluate the efficacy or safety of standalone MMA embolization. Emerging data from observational studies suggest that embolization alone may be a viable therapeutic option in selected patient populations. A recent systematic review and meta-analysis synthesized evidence from eight observational studies, including 156 patients treated with MMA embolization alone and 246 patients treated with combined MMA embolization and surgical evacuation [40]. The analysis revealed low rates of surgical recurrence (i.e., requiring rescue surgery) in both groups, with no statistically significant difference, suggesting that standalone embolization can achieve durable hematoma control in the majority of cases. These findings highlight the potential of embolization as a minimally invasive alternative in patients who are poor surgical candidates, are asymptomatic or mildly symptomatic, or have small-to-moderate hematomas with minimal midline shift. Importantly, subgroup analyses from the MAGIC-MT and STEM trials also suggested a trend toward greater relative benefit of embolization in non-surgical patients [31,32], although these analyses were exploratory and not powered for definitive conclusions. Further RCTs specifically designed to evaluate MMA embolization as a monotherapy are warranted, as this approach could substantially expand treatment options for a growing population of elderly and comorbid patients with cSDH.

Another important clinical consideration is the use of antithrombotic therapy at the time of cSDH diagnosis. In our analysis, 27% of patients in the MMA embolization group and 29% in the BMT-alone group were receiving antithrombotic medications, including antiplatelets and anticoagulants. However, with the exception of the MAGIC-MT trial, the included RCTs did not provide subgroup analyses stratified by antithrombotic use. Notably, however, MAGIC-MT did not identify any significant interaction between antithrombotic therapy and the primary efficacy outcome of recurrence [31]. Given the extracranial nature of the embolization procedure and its theoretically lower bleeding risk profile, MMA embolization may be a particularly attractive option in patients requiring ongoing antithrombotic therapy. Still, as surgical intervention was performed in the majority of patients across both study arms, our meta-analysis does not directly compare MMA embolization to surgery in this context. Future studies are needed to determine whether embolization confers a safety or efficacy advantage in patients on chronic antithrombotic treatment.

Our findings are consistent with prior meta-analyses, though several important distinctions should be emphasized. Unlike previous reviews, we included only published RCTs, thereby minimizing biases inherent in observational designs such as confounding and patient selection. To our knowledge, this is the most comprehensive aggregate data meta-analysis to date exclusively synthesizing data from all currently available RCTs. For example, Kabir et al. included five RCTs and eight observational studies (*n* = 2960) and similarly reported a significant reduction in recurrence without a difference in functional outcomes [41]. More recently, Shankar et al. included only the three largest RCTs (EMBOLISE, MAGIC-MT, STEM), corroborating our findings regarding the efficacy and safety of MMA embolization [42].

Ongoing and upcoming trials may offer additional insights. The ELIMINATE trial (NCT04511572), currently recruiting patients with cSDH in the Netherlands, includes cognitive and quality-of-life measures as part of its secondary outcomes. Moreover, the MEMBRANE trial (NCT04816591) is expected to present its results at the European Stroke Organisation Conference 2025 and may further inform patient selection, procedural timing, and long-term efficacy. As these studies progress, they will help refine the clinical understanding of MMA embolization’s effectiveness and safety, potentially addressing some of the unanswered questions in the present meta-analysis.

Despite its strengths, this meta-analysis has limitations. Although restricted to RCTs, the number of included studies remains modest (*n* = 6), and heterogeneity exists in study design, follow-up duration, and definitions of outcomes. Notably, patient attrition was relatively high in two of the largest trials (15% in STEM and 13% in EMBOLISE) [30,32], complicating the interpretation of long-term outcomes. Additionally, surgical decisions in several trials were made at the discretion of the treating physician rather than based on randomization, introducing potential selection bias. Furthermore, inclusion and exclusion criteria varied across the included RCTs, particularly with respect to hematoma size thresholds, bilaterality, and baseline functional status. For instance, all studies except MAGIC-MT included patients with bilateral hematomas, which may confound the attribution of neurological deficits to a single lesion. Such heterogeneity in patient selection may affect the generalizability of our findings and underscores the need for future trials with harmonized eligibility criteria and standardized reporting. In addition, future RCTs should incorporate standardized neurocognitive assessments and validated quality-of-life instruments to more comprehensively evaluate patient-centered outcomes following MMA embolization. These measures are especially relevant in the elderly cSDH population and will be essential to defining the true long-term benefits of this minimally invasive approach. Finally, our analysis was based on aggregate data rather than individual patient-level data, limiting subgroup analyses and adjustment for potential confounders. Future individual patient data meta-analyses would help overcome these limitations and provide more granular insights into patient-specific treatment effects.

## 5. Conclusions

In conclusion, MMA embolization is a safe and effective intervention for reducing recurrence in patients with cSDH. However, its impact on functional recovery, hematoma thickness reduction, and mortality remains uncertain. Given the promising safety profile, MMA embolization may serve as a valuable adjunct to conventional treatment of patients with cSDH, particularly for preventing recurrence. Future research should identify patient subgroups that would benefit most from MMA embolization, and also focus on longer-term outcomes, cognitive and quality-of-life measures, and refinement of patient selection criteria to better define the role of embolization in the comprehensive management of cSDH.

## Figures and Tables

**Table 1:** Baseline characteristics of studies included in the systematic review and meta-analysis.

Study	Country	Recruitment Period	Inclusion Criteria	Group	Age (Years; Mean ± Standard Deviation)	Female (%)	Use of Antithrombotic Medication (%)	Baseline Thickness of Hematoma (mm; Mean ± Standard Deviation)
**Ng et al. [27]**	France	Apr 2018 to Oct 2018	Symptomatic cSDH requiring surgery	MMA embolization (*n* = 21)	77.4 ± 10.9	43	33	14.7 ± 5.4
				BMT (*n* = 25)	74.7 ± 13.9	36	36	13.6 ± 4.7
**Lam et al. [28]**	Australia	Apr 2021 to Sep 2022	Symptomatic cSDH requiring surgery, >10 mm thickness	MMA embolization (*n* = 16)	64.2	25	19	21.1
				BMT (*n* = 19)	72.4	42	21	20.9
**Debs et al. [29]**	US	Mar 2019 to Nov 2022	Symptomatic cSDH, pre-mRS < 5	MMA embolization (*n* = 17)	66.1 ± 12	29	35	21 ± 9.4
				BMT (*n* = 18)	70.8 ± 9.4	39	39	23 ± 9.2
**Embolise [30]**	US	Dec 2020 to Aug 2023	Symptomatic cSDH, >15 mm thickness or >5 mm midline shift, focal motor deficit	MMA embolization (*n* = 197)	73 ± 11	27	38	21.6 ± 6.3
				BMT (*n* = 203)	71 ± 11.3	27	39	21.4 ± 6.2
**Magic-MT [31]**	China	Mar 2021 to May 2023	Symptomatic non-acute SDH, pre-mRS < 3	MMA embolization (*n* = 360)	68.5 (60–74) *	19	8	22.8 (18.6–27.1) *
				BMT (*n* = 362)	70 (61–75) *	16	7	22.4 (18.5–26.8) *
**Stem [32]**	US France Germany Spain	Nov 2020 to May 2023	Symptomatic cSDH, >10 mm in thickness	MMA embolization (*n* = 149)	72.8 ± 10.4	35	38	18.2 ± 6
				BMT (*n* = 161)	73.4 ± 11.3	26	42	18 ± 6.3

cSDH: chronic subdural hematoma; mRS: modified Rankin Scale; MMA: middle meningeal artery; BMT: best medical treatment. * Median (interquartile range).

**Table 2:** Overview of analyses for the outcomes of interest.

Outcome	Effect Measure	Value (95% CI)	*p*-Value	Prediction Interval	*n* of Studies	i^2^ (p for Cochrane q)
**Primary Efficacy Outcome**						
Recurrence of SDH	RR	0.5 (0.37–0.69)	<0.001	0.33–0.76	6	0% (0.47)
**Secondary Efficacy Outcomes**						
Good functional outcome	RR	1.01 (0.97–1.05)	0.557	0.93–1.1	3	0% (0.557)
Independent ambulation	RR	1.01 (0.99–1.04)	0.222	0.97–1.06	3	0% (0.98)
Hematoma thickness	SMD	−0.1 (−0.3–0)	0.091	−0.5–0.3	4	42% (0.16)
**Primary Safety Outcome**						
All-cause mortality	RR	1.01 (0.42–2.39)	0.988	0.10–9.96	5	44% (0.15)
**Secondary Safety Outcome**						
Adverse events related with MMA embolization	Pooled Rate	1% (0–3%)	N/A	0–29%	2	35% (0.21)

SDH: subdural hematoma; MMA: middle meningeal artery; RR: risk ratio; SMD: standardized mean difference; N/A: not applicable.

## Supplementary Materials

The following supporting information can be downloaded at: https://www.mdpi.com/article/10.3390/jcm14092862/s1, Methods S1: Complete search algorithm used in MEDLINE and Scopus; Table S1: Excluded studies with reasons of exclusion; Table S2: Embolic materials used in the included studies for the middle meningeal artery embolization procedure; Table S3: Definition and follow-up duration for the primary efficacy outcome in the included studies; Table S4: Pooled proportions per arm for each outcome of interest; Figure S1: Forest plots presenting the mean age (in years) among patients treated with middle meningeal artery embolization (MMAe) (A), the mean age (in years) among patients treated with best medical treatment (BMT; (B)), and the standardized mean difference of age (in years) among the patients treated with MMAe versus BMT (C); Figure S2: Forest plots presenting the pooled proportion of female patients among those treated with MMAe (A), the pooled proportion of female patients treated with BMT (B), and the odds ratio of female patients treated with MMAe versus BMT (C); Figure S3: Forest plots presenting the pooled proportion of patients that underwent surgery among those treated with MMAe (A), the pooled proportion of patients that underwent surgery among those treated with BMT (B), and the odds ratio of patients that underwent surgery of those treated with MMAe versus BMT (C); Figure S4: Forest plots presenting the pooled proportion of patients that were receiving antithrombotics prior to index event among those treated with MMAe (A), the pooled proportion of patients that were receiving antithrombotics among those treated with BMT (B), and the odds ratio of patients that were receiving antithrombotics of those treated with MMAe versus BMT (C); Figure S5: Forest plots presenting the mean thickness of hematoma at baseline (in mm) among patients treated with middle meningeal artery embolization (MMAe) (A), the mean thickness of hematoma at baseline (in mm) among patients treated with best medical treatment (BMT; (B)), and the standardized mean difference of thickness of hematoma at baseline (in mm) among the patients treated with MMAe versus BMT (C); Figure S6: Traffic light plot (A) and summary plot (B) presenting the quality assessment of the included randomized-controlled clinical trials (RCTs), using the Cochrane Collaboration tool (RoB 2); Figure S7: Forest plot presenting the rate of adverse events related to the middle meningeal artery embolization (MMAe) in patients receiving MMAe; Figure S8: Funnel plot on the reported rates of recurrence of subdural hematoma (*p* for Egger’s test = 0.348); Figure S9: Funnel plot on the reported thickness of subdural hematoma at follow-up (*p* for Egger’s test = 0.649); Figure S10: Funnel plot on the reported rates of all-cause mortality (*p* for Egger’s test = 0.288).
