# Supplementary material for: Embolization of Middle Meningeal Artery in Patients with Chronic Subdural Hematoma: A Systematic Review and Meta-Analysis of Randomized-Controlled Clinical Trials
Source: J Clin Med. 2025 Apr 22;14(9):2862. doi: 10.3390/jcm14092862 (PMC12072903; doi:10.3390/jcm14092862)

Supplement:

Methods S1

Tables S1–4

Figures S1–10

Methods S1

Complete search algorithm used in MEDLINE (using PubMed).

("Embolization"[Mesh] OR "Embolization, Therapeutic"[Mesh] OR "Endovascular procedures"[Mesh] OR "Arterial embolization"[Mesh]) AND ("Middle meningeal artery"[Mesh] OR "Meningeal arteries"[Mesh] OR "Middle meningeal artery embolization") AND ("Chronic subdural hematoma"[Mesh] OR "Chronic subdural hematoma"[Text Word] OR "Subdural hematoma, chronic"[Text Word]) AND ("Interventional radiology"[Mesh] OR "Endovascular treatment"[Mesh] OR "Outcome")

Complete search algorithm used in Scopus.

TITLE-ABS-KEY ("emboliz*" OR "therapeutic embolization" OR "endovascular procedures" OR "arterial embolization") AND TITLE-ABS-KEY ("middle meningeal artery" OR "meningeal arteries" OR "middle meningeal artery embolization") AND TITLE-ABS-KEY ("chronic subdural hematoma" OR "chronic subdural hematoma surgery" OR "subdural hematoma, chronic") AND TITLE-ABS-KEY ("interventional radiology" OR "endovascular treatment" OR "outcome")

Table S1: Excluded studies with reasons of exclusion.

Study name	Reason for exclusion
Onyinzo et al. 2021	Wrong study design
Marulanda et al. 2021	Wrong study design
Catapano et al. 2021	Wrong study design
Shotar et al. 2020	Wrong study design
Carpenter et al. 2021	Wrong study design
Ban et al. 2018	Wrong study design
Matsumoto et al. 2018	Wrong study design
Kim et al. 2017	Wrong study design

Table S2: Embolic materials used in the included studies for the middle meningeal artery embolization procedure.

STUDY	EMBOLIC MATERIALS
**Ng et al.**	polyvinyl alcohol particles (150–250 µm diameter)
**Lam et al.**	Squid-12 (Balt, Montmorency, France), Onyx-18 (Medtronic, Irvine, CA, USA), Phil 25% (MicroVention, Aliso Viejo, USA), and 25% n-butyl cyanoacrylate (n-BCA) (B. Braun, Melsungen, Germany) with 75% Lipiodol (Guerbet, Villepinte, France), at the discretion of the treating neurointerventionist
**Debs et al.**	liquid embolic agent Ethylene Vinyl Alcohol Copolymer (Onyx; Medtronic, Irvine, CA, USA)
**EMBOLISE**	liquid embolic agent Ethylene Vinyl Alcohol Copolymer (Onyx; Medtronic, Irvine, CA, USA)
**MAGIC-MT**	liquid embolic agent Ethylene Vinyl Alcohol Copolymer (Onyx; Medtronic, Irvine, CA, USA)
**STEM**	liquid embolic agent Ethylene Vinyl Alcohol Copolymer (Squid; Balt, Montmorency, France)

Table S3: Definition and follow-up duration for the primary efficacy outcome in the included studies.

Study	Definition of primary efficacy outcome	Follow-up period
**Ng et al.**	Persistent subdural collection on brain CT with persistent or new symptoms	90 days
**Lam et al.**	persistent or new cSDH on brain CT with persistent or new symptoms, requiring surgery	90 days
**Debs et al.**	New or recurrent symptoms, requiring surgery	125 days
**EMBOLISE**	Presence of imaging evidence of SDH with or without new or worsening symptoms, requiring surgery	90 days
**MAGIC-MT**	SDH thickness exceeding 10 mm or reoperation during follow-up, or increase in the SDH thickness by more than 3 mm from baseline	90 days
**STEM**	SDH greater than 10 mm or requiring surgery	180 days

cSDH: chronic subdural hematoma

Table S4: Pooled proportions per arm for each outcome of interest.

Outcomes of interest	Pooled Proportion (95% Confidence Interval)	
	MMA embolization	BMT
**Primary Efficacy Outcome**		
Recurrence of SDH	6% (3-10%)	17% (9-27%)
**Secondary Efficacy Outcomes**		
Good functional outcome	87% (78-94%)	86% (76-93%)
Independent Ambulation	97% (95-99%)	96% (93-98%)
Thickness of hematoma at follow-up	5.3 (4.9-5.7) *	6.4 (5.0-7.7) *
**Primary Safety Outcome**		
All-cause mortality	2% (0-7%)	3% (2-5%)

SDH: Subdural Hematoma

* mm (95%CI)

Figure S1. Forest plots presenting the mean age (in years) among patients treated with middle meningeal artery embolization (MMAe) (**A**), the mean age (in years) among patients treated with best medical treatment (BMT; (**B**)), and the standardized mean difference of age (in years) among the patients treated with MMAe versus BMT (**C**).

A.

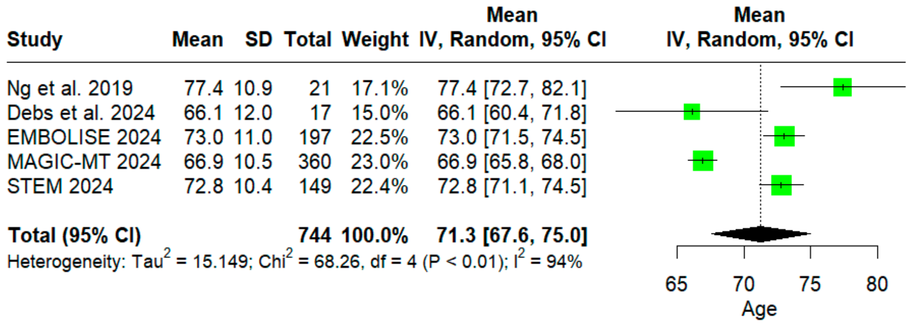

B.

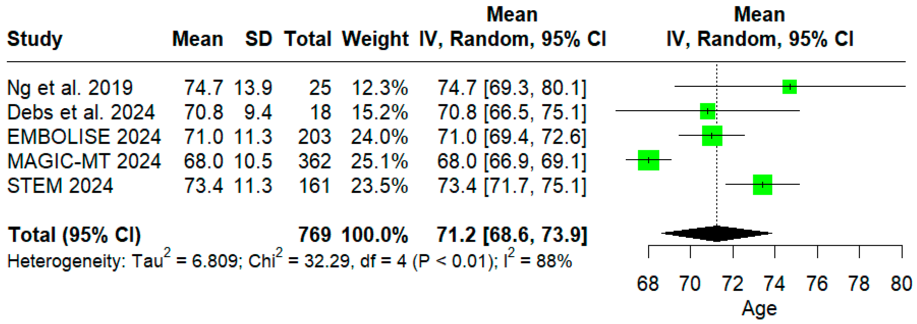

C.

Study	MMAe Mean	MMAe SD	MMAe Total	BMT Mean	BMT SD	BMT Total	Weight	Std. Mean Difference IV, Random, 95% CI
Ng et al. 2019	77.4	10.9	21	74.7	13.9	25	6.5%	0.2 [-0.4, 0.8]
Debs et al. 2024	66.1	12.0	17	70.8	9.4	18	5.1%	-0.4 [-1.1, 0.2]
EMBOLISE 2024	73.0	11.0	197	71.0	11.3	203	28.3%	0.2 [-0.0, 0.4]
MAGIC-MT 2024	66.9	10.5	360	68.0	10.5	362	34.9%	-0.1 [-0.3, 0.0]
STEM 2024	72.8	10.4	149	73.4	11.3	161	25.2%	-0.1 [-0.3, 0.2]
**Total (95% CI)**			**744**			**769**	**100.0%**	**-0.0 [-0.2, 0.2]**

Heterogeneity: $Tau^2 = 0.013$; $Chi^2 = 7.34$, df = 4 (P = 0.12); $I^2 = 46\%$

Test for overall effect: Z = -0.10 (P = 0.924)

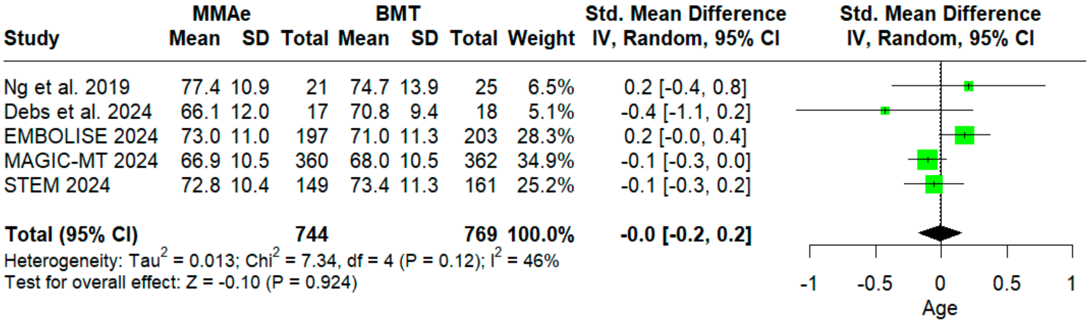

Figure S2. Forest plots presenting the pooled proportion of female patients among those treated with MMAe (A), the pooled proportion of female patients treated with BMT (B), and the odds ratio of female patients treated with MMAe versus BMT (C).

A.

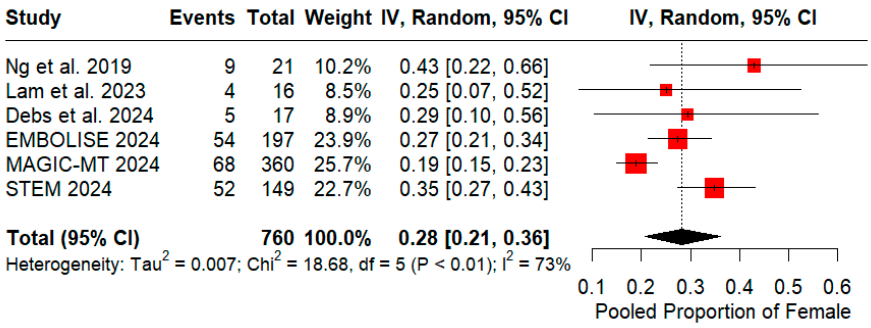

B.

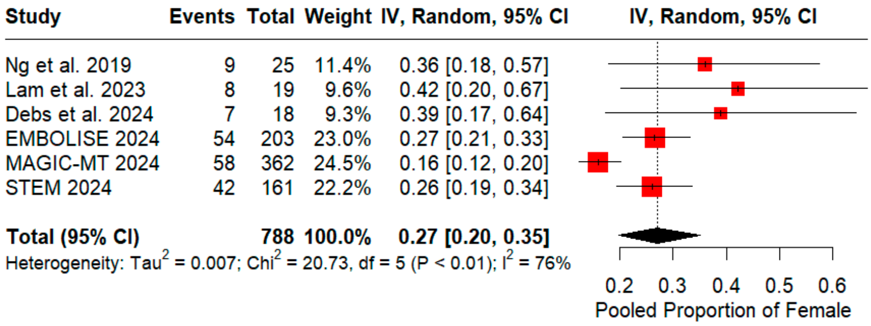

C.

Study	MMAe Events	MMAe Total	BMT Events	BMT Total	Weight	Odds Ratio IV, Random, 95% CI
Ng et al. 2019	9	21	9	25	4.0%	1.3 [0.4, 4.4]
Lam et al. 2023	4	16	8	19	2.7%	0.5 [0.1, 2.0]
Debs et al. 2024	5	17	7	18	2.8%	0.7 [0.2, 2.7]
EMBOLISE 2024	54	197	54	203	28.9%	1.0 [0.7, 1.6]
MAGIC-MT 2024	68	360	58	362	37.9%	1.2 [0.8, 1.8]
STEM 2024	52	149	42	161	23.7%	1.5 [0.9, 2.5]
**Total (95% CI)**		**760**		**788**	**100.0%**	**1.2 [0.9, 1.5]**

Heterogeneity: $Tau^2 = 0$; $Chi^2 = 3.71$, df = 5 (P = 0.59); $I^2 = 0\%$
Test for overall effect: Z = 1.37 (P = 0.172)

Odds Ratio
IV, Random, 95% CI

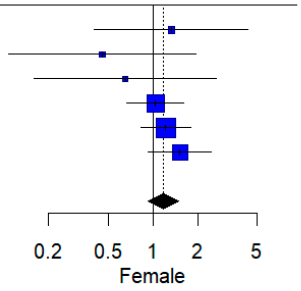

Figure S3. Forest plots presenting the pooled proportion of patients that underwent surgery among those treated with MMAe (A), the pooled proportion of patients that underwent surgery among those treated with BMT (B), and the odds ratio of patients that underwent surgery of those treated with MMAe versus BMT (C).

A.

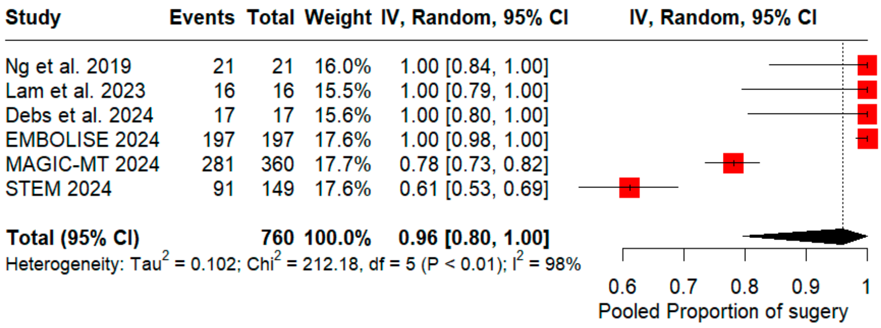

B.

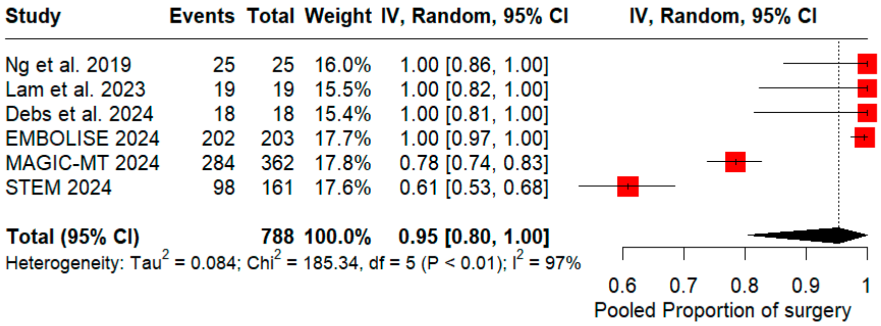

C.

Study	MMAe Events	MMAe Total	BMT Events	BMT Total	Weight	Odds Ratio IV, Random, 95% CI
Ng et al. 2019	21	21	25	25	0.0%	
Lam et al. 2023	16	16	19	19	0.0%	
Debs et al. 2024	17	17	18	18	0.0%	
EMBOLISE 2024	197	197	202	203	0.8%	2.9 [0.1, 72.3]
MAGIC-MT 2024	281	360	284	362	62.0%	1.0 [0.7, 1.4]
STEM 2024	91	149	98	161	37.2%	1.0 [0.6, 1.6]
**Total (95% CI)**		**760**		**788**	**100.0%**	**1.0 [0.8, 1.3]**

Heterogeneity: $Tau^2 = 0$; $Chi^2 = 0.45$, df = 2 (P = 0.80); $I^2 = 0\%$
Test for overall effect: Z = -0.02 (P = 0.982)

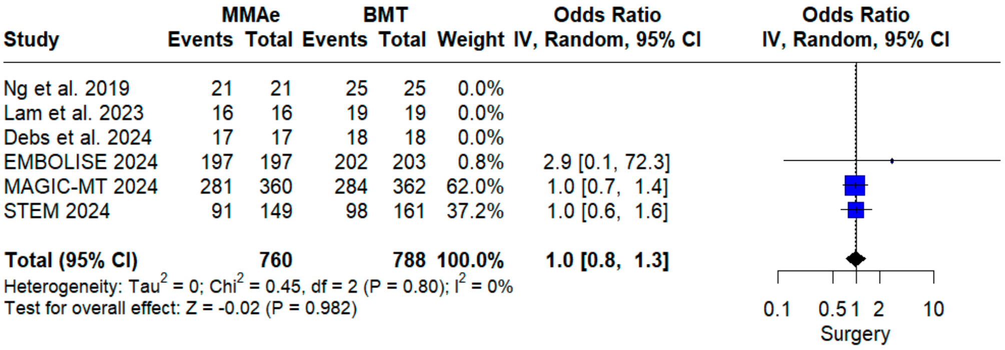

Figure S4. Forest plots presenting the pooled proportion of patients that were receiving antithrombotics prior to index event among those treated with MMAe (A), the pooled proportion of patients that were receiving antithrombotics among those treated with BMT (B), and the odds ratio of patients that were receiving antithrombotics of those treated with MMAe versus BMT (C).

A.

Study	Events	Total	Weight	IV, Random, 95% CI
Ng et al. 2019	7	21	15.3%	0.33 [0.15, 0.57]
Lam et al. 2023	3	16	14.4%	0.19 [0.04, 0.46]
Debs et al. 2024	6	17	14.6%	0.35 [0.14, 0.62]
EMBOLISE 2024	75	197	18.5%	0.38 [0.31, 0.45]
MAGIC-MT 2024	28	360	18.7%	0.08 [0.05, 0.11]
STEM 2024	56	149	18.4%	0.38 [0.30, 0.46]
**Total (95% CI)**		**760**	**100.0%**	**0.27 [0.12, 0.45]**

Heterogeneity: $Tau^2 = 0.050$; $Chi^2 = 105.56$, df = 5 ($P < 0.01$); $I^2 = 95\%$

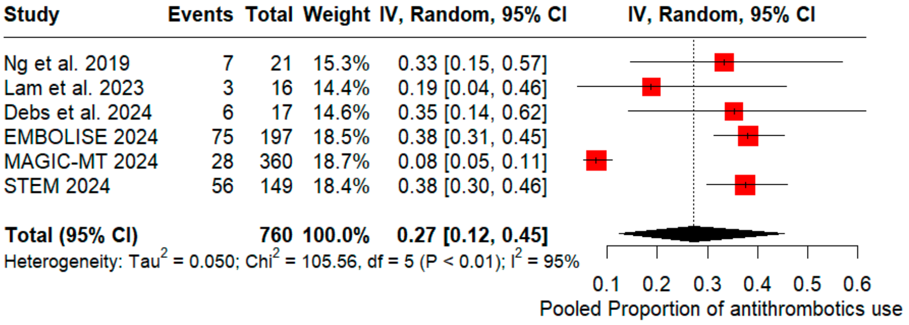

B.

Study	Events	Total	Weight	IV, Random, 95% CI
Ng et al. 2019	9	25	15.8%	0.36 [0.18, 0.57]
Lam et al. 2023	4	19	15.1%	0.21 [0.06, 0.46]
Debs et al. 2024	7	18	14.9%	0.39 [0.17, 0.64]
EMBOLISE 2024	79	203	18.0%	0.39 [0.32, 0.46]
MAGIC-MT 2024	25	362	18.2%	0.07 [0.05, 0.10]
STEM 2024	67	161	17.9%	0.42 [0.34, 0.50]
**Total (95% CI)**		**788**	**100.0%**	**0.29 [0.13, 0.49]**

Heterogeneity: $Tau^2 = 0.060$; $Chi^2 = 133.55$, df = 5 ($P < 0.01$); $I^2 = 96\%$

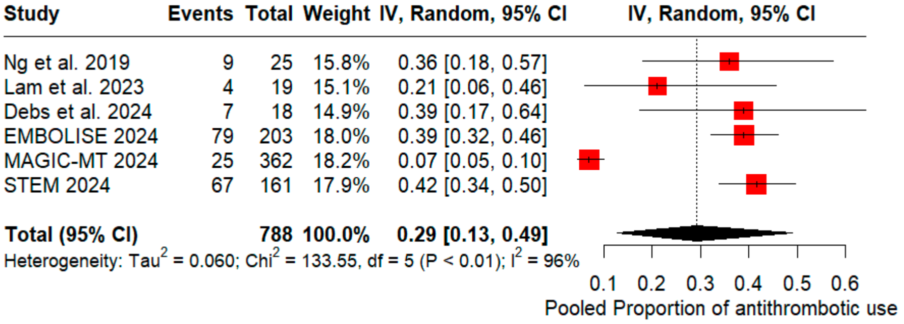

C.

Study	MMAe Events	MMAe Total	BMT Events	BMT Total	Weight	Odds Ratio IV, Random, 95% CI
Ng et al. 2019	7	21	9	25	4.3%	0.9 [0.3, 3.0]
Lam et al. 2023	3	16	4	19	2.3%	0.9 [0.2, 4.6]
Debs et al. 2024	6	17	7	18	3.4%	0.9 [0.2, 3.4]
EMBOLISE 2024	75	197	79	203	39.2%	1.0 [0.6, 1.4]
MAGIC-MT 2024	28	360	25	362	20.3%	1.1 [0.6, 2.0]
STEM 2024	56	149	67	161	30.6%	0.8 [0.5, 1.3]
**Total (95% CI)**		**760**		**788**	**100.0%**	**0.9 [0.7, 1.2]**

Heterogeneity: $Tau^2 = 0$; $Chi^2 = 0.70$, df = 5 (P = 0.98); $I^2 = 0\%$
Test for overall effect: Z = -0.41 (P = 0.680)

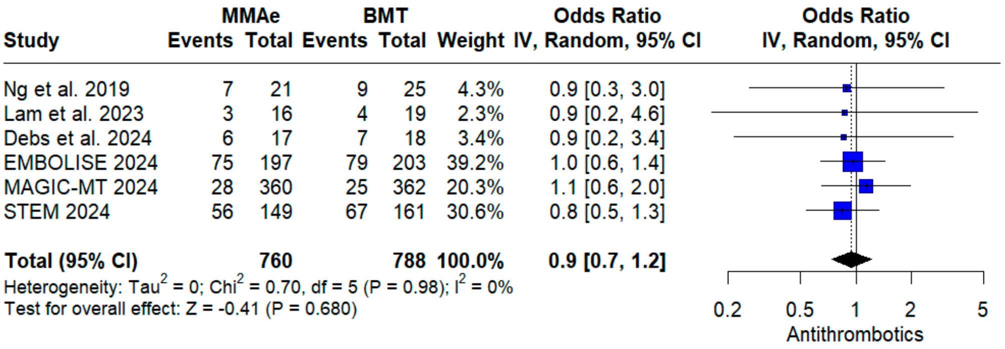

Figure S5. Forest plots presenting the mean thickness of hematoma at baseline (in mm) among patients treated with middle meningeal artery embolization (MMAe) (A), the mean thickness of hematoma at baseline (in mm) among patients treated with best medical treatment (BMT; (B)), and the standardized mean difference of thickness of hematoma at baseline (in mm) among the patients treated with MMAe versus BMT (C).

A.

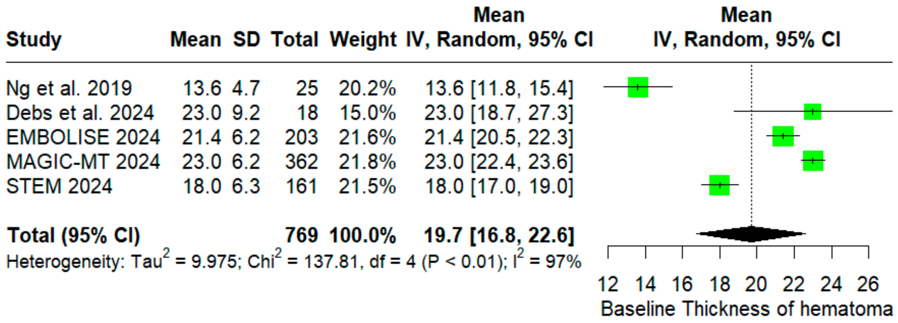

B.

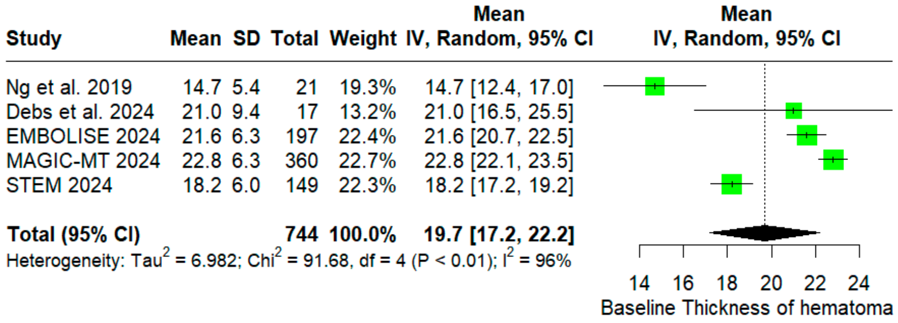

C.

Study	MMAe Mean	MMAe SD	MMAe Total	BMT Mean	BMT SD	BMT Total	Weight	Std. Mean Difference IV, Random, 95% CI
Ng et al. 2019	14.7	5.4	21	13.6	4.7	25	3.0%	0.2 [-0.4, 0.8]
Debs et al. 2024	21.0	9.4	17	23.0	9.2	18	2.3%	-0.2 [-0.9, 0.5]
EMBOLISE 2024	21.6	6.3	197	21.4	6.2	203	26.5%	0.0 [-0.2, 0.2]
MAGIC-MT 2024	22.8	6.3	360	23.0	6.2	362	47.8%	-0.0 [-0.2, 0.1]
STEM 2024	18.2	6.0	149	18.0	6.3	161	20.5%	0.0 [-0.2, 0.3]
**Total (95% CI)**			**744**			**769**	**100.0%**	**0.0 [-0.1, 0.1]**

Heterogeneity: $Tau^2 = 0$; $Chi^2 = 1.27$, df = 4 (P = 0.87); $I^2 = 0\%$
Test for overall effect: Z = 0.03 (P = 0.978)

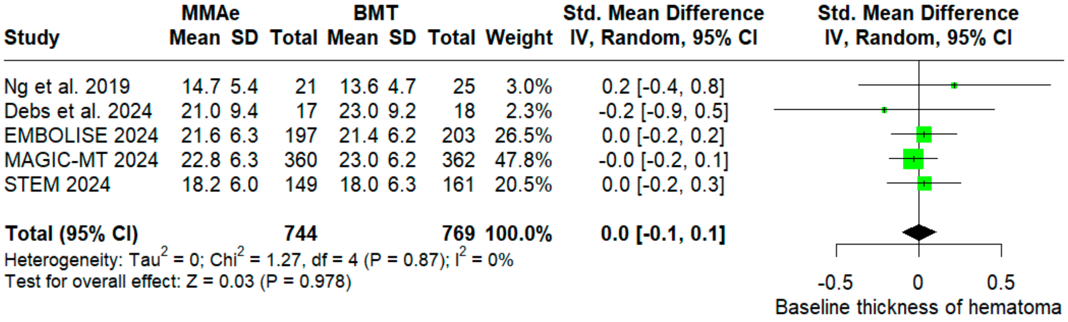

Figure S6. Traffic light plot (A) and summary plot (B) presenting the quality assessment of the included randomized-controlled clinical trials (RCTs), using the Cochrane Collaboration tool (RoB 2).

A.

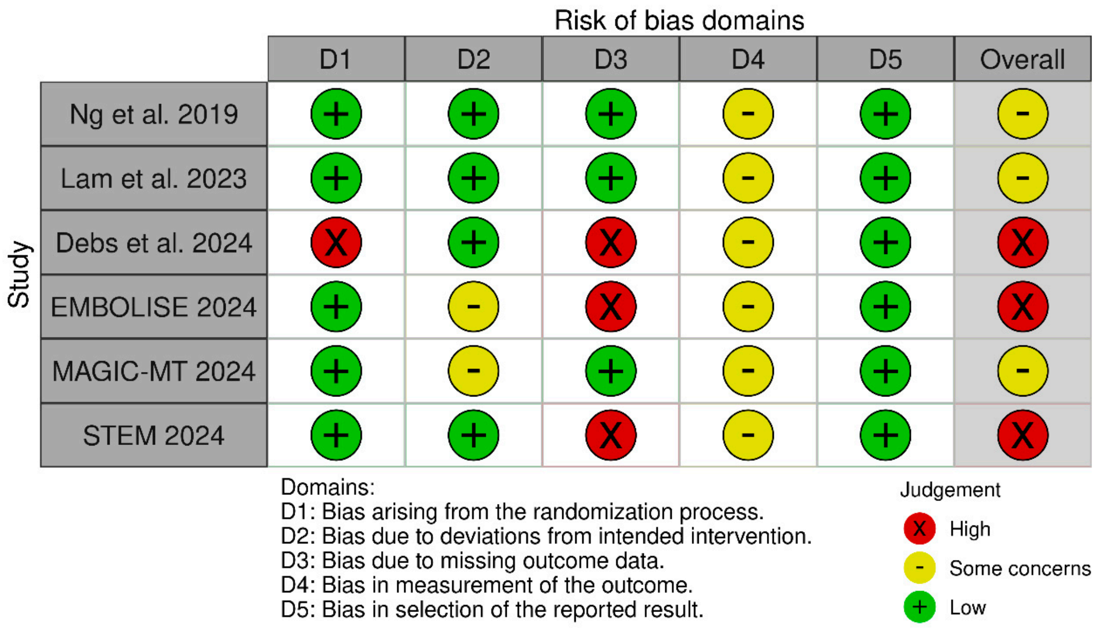

B.

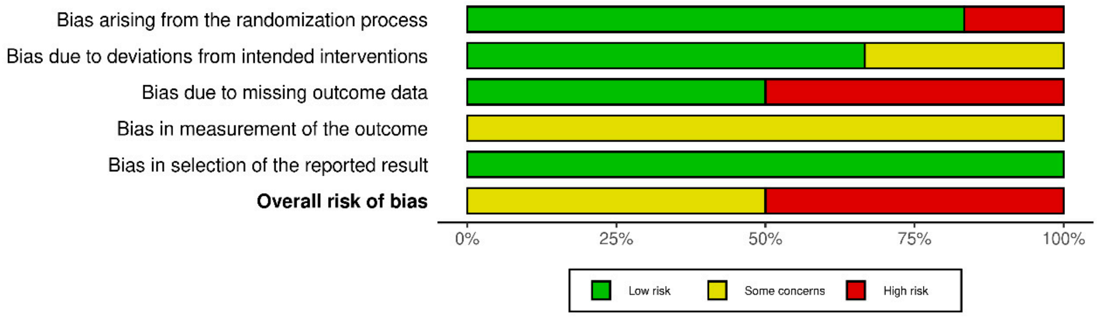

Figure S7: Forest plot presenting the rate of adverse events related to the middle meningeal artery embolization (MMAe) in patients receiving MMAe.

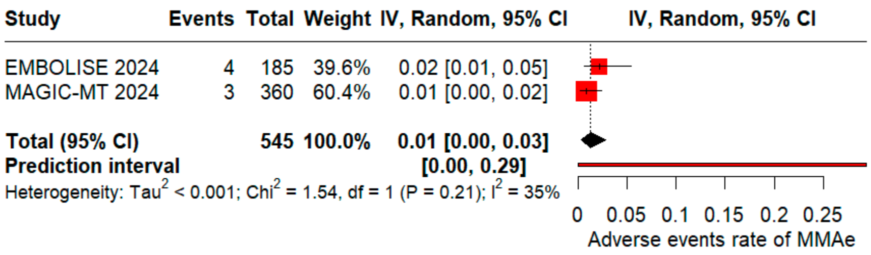

Figure S8. Funnel plot on the reported rates of recurrence of subdural hematoma (p for Egger's test= 0.348).

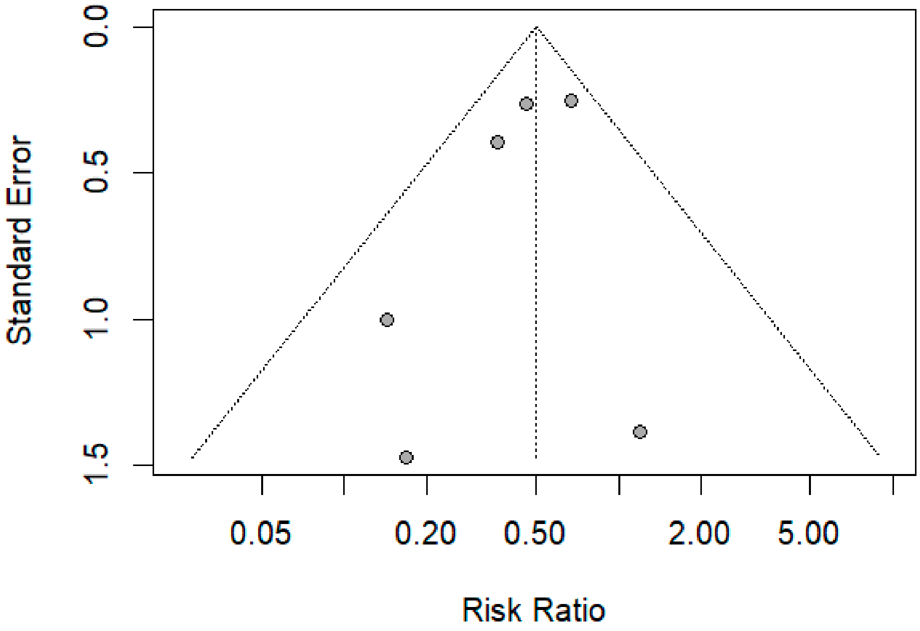

Figure S9. Funnel plot on the reported thickness of subdural hematoma at follow-up (p for Egger's test= 0.649).

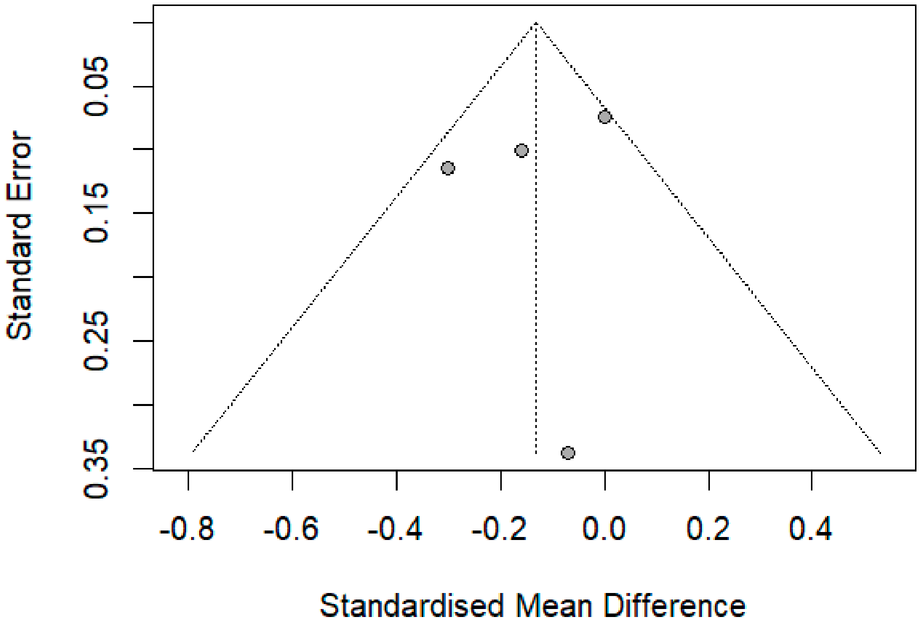

Figure S10. Funnel plot on the reported rates of all-cause mortality (p for Egger's test= 0.288).

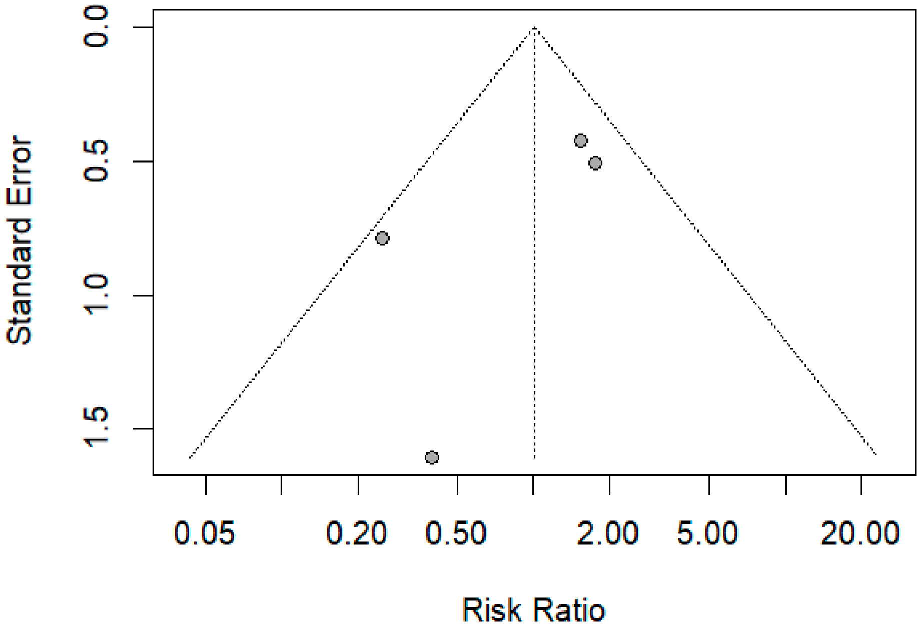

Supplement: Supplementary file 1 [file jcm-14-02862-s001.zip › jcm-3589831-supplementary.pdf]
